# Correlation of Serum Prolactin Levels With Chronic Liver Disease Severity in a Tertiary Care Hospital in Eastern India

**DOI:** 10.7759/cureus.77164

**Published:** 2025-01-08

**Authors:** Sangita D Kamath, Alipa Sinha, Neelam Mehta, Rakesh Singh

**Affiliations:** 1 Internal Medicine, Tata Main Hospital, Jamshedpur, IND; 2 Biochemistry, Tata Main Hospital, Jamshedpur, IND

**Keywords:** child-turcotte-pugh score, complications, disease, encephalopathy, liver, portal hypertension, : prolactin

## Abstract

Introduction

Chronic liver disease (CLD) is a health concern in India and is associated with significant morbidity and mortality. Hormonal dysregulation, including impaired, hepatic elimination of hormones, is a common feature of CLD. Prolactin, a hormone traditionally associated with reproductive functions, is a potential biomarker for assessing the severity and complications of CLD. This study aimed to evaluate the relationship between serum prolactin levels and the severity of CLD, as assessed by the Child-Turcotte-Pugh (CTP) score, and to explore its association with various complications of the disease.

Methods

This prospective observational study was conducted in the Department of Medicine at Tata Main Hospital, Jamshedpur, India, from June 2022 to December 2023. Patients diagnosed with CLD were evaluated for demographic characteristics, clinical history, and examination findings. Comprehensive diagnostic work-ups for CLD and its complications were performed, and disease severity was assessed using the CTP score. Serum prolactin levels were measured using an electrochemiluminescence assay. Statistical analyses, including the Chi-square test, Kruskal-Wallis test, and independent sample t-tests, were employed to evaluate the relationship between serum prolactin levels, disease severity, and complications. A p-value of <0.05 was considered statistically significant.

Results

The study included 120 patients with a mean age of 47.3 ± 10.4 years. There was a marked male predominance, with a male-to-female ratio of 6.5:1. Alcohol consumption was the most common cause of CLD (n=75, 62.5%), followed by metabolic dysfunction-associated steatohepatitis (n=11, 9.2%) and hepatitis B infection (n=10, 8.3%). The mean serum prolactin level was 35.3 ±, 9.25 ng/mL (range: 16 to 52 ng/mL), and the mean CTP score was 9.68 ±, 2.11. Most patients were classified as CTP Class B (n=31, 51.7%), followed by Class C (n=55, 45.8%) and Class A (n=3, 2.5%).

Serum prolactin levels showed significant positive correlations with CTP scores, serum bilirubin (R = 0.377, p < 0.001), and international normalized ratio (R = 0.317, p < 0.001) and a significant negative correlation with serum albumin (R = -0.393, p < 0.001). The Kruskal-Wallis test demonstrated a significant association between serum prolactin levels and the severity of ascites (p < 0.001) and hepatic encephalopathy (p < 0.001). Using receiver operating characteristic analysis, a serum prolactin cut-off > 35 ng/mL predicted severe CLD (CTP Class C) with 83.6% sensitivity and 83.1% specificity. Elevated serum prolactin levels were observed in 77.4% of patients (n=60) with oesophageal varices, 88.2% (n=30) with upper gastrointestinal bleeding, 93.7% (n=39) with hepatic encephalopathy, 75.5% (n=53) with moderate to severe ascites, and 82.8% (n=3) with spontaneous bacterial peritonitis. The overall mortality rate was 3.33% (n=4), and serum prolactin levels were significantly higher in deceased patients compared to those in survivors (p < 0.001).

Conclusion

Serum prolactin levels strongly correlate with the severity of CLD as measured by the CTP scoring system. Our results support the potential utility of serum prolactin as a prognostic biomarker for complications and outcomes in CLD. Its non-invasive nature and significant association with disease severity and complications make it a valuable clinical assessment and management tool.

## Introduction

Chronic liver disease (CLD) is a progressive condition characterized by inflammation, disruption of liver architecture, fibrosis, and regeneration of liver parenchyma, ultimately leading to cirrhosis. It is a significant cause of morbidity and mortality in India and worldwide. A meta-analysis and review of the etiological spectrum of liver cirrhosis in adults in the Indian population by Swaroop et al. reported that alcohol was associated with cirrhosis in 43.2% of cases. In comparison, metabolic dysfunction-associated steatotic liver disease accounted for 14.4%. Additionally, hepatitis B virus and hepatitis C virus infections contributed to 11.5% and 6.2% of cases, respectively [1]. Liver disease accounted for 2.95% of total deaths in India and one-fifth (18.3%) of all cirrhosis deaths globally [2]. In contrast to China, the burden of CLD and cirrhosis in India has been steadily rising since 1980 [3]. These diseases often present at advanced stages, with clinical decompensation limiting opportunities for curative treatment.

Patients with CLD exhibit altered metabolic, immunological, synthetic, and hormonal functions. Hormonal dysregulation results from impaired hormone elimination, altered secretion, and defective feedback mechanisms [4]. One hormone of interest is prolactin, traditionally associated with reproductive functions and lactation. Prolactin is secreted by anterior pituitary lactotrophs, with its release inhibited by dopamine from the hypothalamus via the tuberoinfundibular tract and stimulated by hypothalamic releasing factors and circulating oestrogens [5]. In CLD, serum oestrogen levels are elevated due to increased aromatization of testosterone in peripheral tissues and reduced hepatic clearance. Elevated oestrogen levels suppress hypothalamic dopamine release, stimulating prolactin secretion, and directly act on the anterior pituitary, contributing to hyperprolactinemia. The synthesis of false neurotransmitters, such as octopamine and phenylethanolamine, from aromatic amino acids is increased in CLD, further inhibiting dopamine release and exacerbating hyperprolactinemia [6].

The Child-Turcotte-Pugh (CTP) score is an established tool for assessing the severity of liver dysfunction and predicting outcomes in CLD. It incorporates clinical parameters, such as encephalopathy and ascites, and biochemical measures, including serum albumin, serum bilirubin, and prothrombin time/international normalized ratio (INR). Each parameter is scored from 1 to 3, with the total score categorizing hepatic dysfunction as grade A (mild), B (moderate), or C (severe) [7]. However, the subjective nature of encephalopathy and ascites assessment and the exclusion of renal function limit the accuracy of the CTP score in predicting prognosis.

Given that hormonal disturbances occur in advanced liver dysfunction and that serum prolactin levels increase in CLD, prolactin may serve as a readily available biomarker to assess disease severity and provide prognostic insight. Few studies in India have explored the association of serum prolactin with the severity of CLD, and data from Eastern India remains limited. In this context, we conducted a study to assess the relationship between serum prolactin levels and the severity of CLD, as measured by the CTP score, and to evaluate the association of serum prolactin levels with various complications of CLD.

## Materials and methods

This prospective observational study included patients seen in the outpatient and inpatient departments of Medicine and Gastroenterology at Tata Main Hospital (TMH) in Jamshedpur, Jharkhand, India from June 1, 2022, to December 31, 2023. TMH is a 983-bed multidisciplinary industrial hospital. The Institutional Ethics Committee approved the study (Approval No. TMH/IEC/JUNE/053/22), and all participants provided informed consent.

We defined CLD as hepatic dysfunction persisting for six months or longer. Patients with CLD exhibited signs such as splenomegaly, abnormal international normalised ratio (INR), hypoalbuminemia, jaundice, ascites, varices, or ultrasound evidence of CLD upon admission. The study included patients aged 18 years or older with a diagnosis of CLD and its complications who consented to participate. Patients were excluded if they had a history of pituitary, hypothalamic, or thyroid disease; chronic renal failure; herpes zoster; cranial surgery or irradiation; or chest wall trauma. Other exclusion criteria included pregnancy, lactation, and the use of medications known to elevate prolactin levels, such as neuroleptics, metoclopramide, methyldopa, reserpine, and cyproterone acetate.

The sample size was calculated using the formula:

\[ n = \frac{[Z_{1-\alpha/2} + Z_{1-\beta}]^2 + 3}{C^2} \]

Where, \[ C = \frac{1}{2} \ln \left( \frac{1+r}{1-r} \right) \]

Interpretation of Notation

$Z_{1-\alpha/2}$ = Value of the standard normal deviate at 95% level of confidence interval $Z_{1-\beta}$ = Value of the standard normal deviate at 80% power of the study $r$ = Correlation between serum prolactin and Child-Pugh class $C$ = Fisher Z transformation of the minimum correlation coefficient to be detected

Estimation of Parameters From the Previous/Pilot Study

$Z_{1-\alpha/2}$ = 1.96 $Z_{1-\beta}$ = 0.84 (80% power) $r$ = 0.079 $C$ = 0.079

Total Sample Size (n) = 120 (after consideration of 10% dropout)

Data collection included demographic information such as age, gender, presenting symptoms, and clinical findings. We documented the presence of cirrhosis and its complications, including ascites, portal hypertension, oesophageal varices, hepatic encephalopathy (HE), hepatorenal syndrome, and spontaneous bacterial peritonitis [8]. We also recorded the medical history of comorbidities, alcohol use, other addictions, medication use, and family history.

Participants underwent comprehensive diagnostic evaluations for CLD and its complications. Laboratory tests included complete blood count, urinalysis, renal function tests (blood urea and serum creatinine), liver function tests (serum bilirubin, alanine transaminase, aspartate transaminase, alkaline phosphatase, total serum proteins, albumin, and globulin levels, prothrombin time, and INR), and ascitic fluid analysis (glucose, protein, lactate dehydrogenase, cytology, and microbiological cultures). Imaging studies included abdominal ultrasound to assess liver echotexture and size, splenic enlargement, portal vein diameter, and Doppler studies of the hepatic and portal veins to detect thrombosis. An endoscopy was performed to identify oesophageal varices, portal hypertensive gastropathy, or duodenopathy.

Viral markers, including hepatitis B surface antigen and anti-hepatitis C virus antibodies, were assessed using enzyme-linked immunosorbent assay. Additional investigations were conducted in selected cases. Tests for autoimmune and metabolic causes of liver disease included serum ceruloplasmin, slit-lamp examination, and autoimmune hepatitis panels, which included anti-nuclear antibody, anti-smooth muscle antibody, anti-liver kidney muscle antibody, and anti-mitochondrial antibody. Serum prolactin levels were measured at admission using a chemiluminescence immunoassay kit provided by Roche Diagnostics (Indianapolis, IN, USA).

The severity of liver dysfunction was classified using the modified CTP system. This system categorizes patients into Class A (5 to 6 points, compensated disease), Class B (7 to 9 points, decompensated disease), and Class C (10 to 15 points, severe, decompensated disease; Table 1). Clinical definitions for HE were based on the West Haven classification system (Table 2). Ascites was graded as mild, moderate, or large according to the International Club of Ascites [9]. Mild ascites was detected only by ultrasound, moderate ascites caused symmetrical abdominal distension, and large ascites was associated with marked abdominal distension. Hepatorenal syndrome was diagnosed using the revised consensus recommendations of the International Club of Ascites [10].

**Table 1:** Modified CTP scoring system Abbreviations: CTP, Child-Turcotte-Pugh; INR, international normalized ratio. Based on Tstoris and Marlar [7]

Clinical and Laboratory Criteria	Points		
	1	2	3
Encephalopathy	None	Mild to Moderate (Grade 1 or 2)	Severe (Grade 3 or 4)
Ascites	None	Mild to Moderate (Diuretic Responsive)	Severe (Diuretic Refractory)
Bilirubin (mg/dl)	<2	2-3	>3
Albumin (g/dl)	>3.5	2.8-3.5	<2.8
Prothrombin Time (seconds prolonged)	<4	4-6	>6
INR	<1.7	1.7-2.3	>2.3

**Table 2:** West Haven grading of HE Abbreviation: HE, hepatic encephalopathy Based on Weissenborn [8]

Grade	Clinical definition for HE
Grade1 (covert)	Decreased attention span, mild personality changes, euphoria/anxiety, altered sleep pattern
Grade 2	Lethargy, disorientation in time, asterixis, hyporeflexia, speech abnormalities
Grade 3	Somnolence to stupor, asterixis, place and time disorientation, hyperreflexia, Babinski reflex positive
Grade 4	Coma (nonresponsive to painful stimuli)

Spontaneous bacterial peritonitis was defined as a bacterial infection of ascitic fluid, with or without positive cultures, and an elevated polymorphonuclear neutrophil count of >250/mm³ in the absence of an identifiable treatable intra-abdominal infection [11]. Hepatopulmonary syndrome was defined as hypoxemia, indicated by an oxygen saturation of ≤80 mmHg while breathing room air or an alveolar-arterial oxygen gradient of ≥15 mmHg, due to intrapulmonary vasodilation in the presence of portal hypertension [12]. Normal serum prolactin levels were defined as 10 to 20 ng/mL for men and 10 to 25 ng/mL for nonpregnant women [13].

Statistical analysis 

Data were analysed using IBM SPSS Statistics for Windows, Version 22.0. (Armonk, NY: IBM Corp.). Frequencies and proportions were used to describe categorical data, while means and standard deviations were calculated for continuous variables. As appropriate, statistical significance was determined using the Chi-square test for categorical data and the independent t-test or Kruskal-Wallis test for continuous variables. Pearson’s correlation coefficient was calculated to evaluate relationships between continuous variables. A p-value < 0.05 was considered statistically significant. Receiver operating characteristic (ROC) curve analysis was performed to evaluate the ability of serum prolactin levels to predict severe CLD (CTP Class C). The area under the ROC curve (AUC), 95% confidence interval (CI), p-value, and the optimal prolactin cut off level were calculated to assess predictive accuracy.

## Results

The study included 120 cases of CLD observed between June 2022 and December 2023 at the Department of Medicine, Tata Main Hospital. The mean age of the study population was 47.3 ± 10.3 years, with most patients (69.2%, n=83) falling within the 40- to 59-year age group. The ages ranged from 19 to 73 years (Table 3). There was a clear male preponderance, with 104 men (86.7%) and 16 women (13.3%), resulting in a male-to-female ratio of 6.5:1. The mean weight of participants was 62.3 ± 8.75 kg, and the mean body mass index (BMI) was 23.4 ± 3.9 kg/m². Most patients (n=70, 58.3%) had a BMI between 18.5 kg/m² and 24.9 kg/m², while five (4.2%) were morbidly obese, and 10 (8.3%) had a BMI of less than 18.5 kg/m².

**Table 3:** Age distribution in study population (n=120)

Age Range Group (Years)	Number	Percentage
18-20	2	1.7%
20-29	3	2.5%
30-39	19	15.8%
40-49	42	35%
50-59	41	34.2%
60-69	12	10%
70-75	1	0.8%
Total	120	100%

Alcoholism emerged as the most common cause of CLD, affecting 75 patients (62.5%), followed by metabolic dysfunction-associated steatohepatitis (MASH) in 11 (9.2%) and hepatitis B virus infection in 10 (8.3%). Cryptogenic causes accounted for nine cases (7.5%). The distribution of etiological factors is presented in Table 4.

**Table 4:** Etiology of CLD in the study population (n=120) Abbreviations: CLD, chronic liver disease; HBV, hepatitis B virus; HCV, hepatitis C virus; MASH, metabolic syndrome-related steatohepatitis.

Cause of CLD	Number	Percentage
Alcohol	75	62.5%
Alcohol + HBV	3	2.5%
Autoimmune	3	2.5%
Biliary cholangitis	3	2.5%
Cryptogenic	9	7.5%
HBV	10	8.3%
HCV	6	5%
MASH	11	9.2%

Generalized weakness and abdominal distension were the most common clinical features, present in 90.8% (n=109) and 88.3% (n=106) of patients, respectively. Other symptoms included easy bruising (n=97, 80.8%), fatigue (n=63, 52.5%), loss of appetite and nausea (n=58, 48.3%), jaundice (n=48, 40%), and altered sensorium (n=30, 25%), as shown in Table 5.

**Table 5:** Distribution of symptoms (n=120)

Clinical features	Number of cases	Percentage of cases
Generalized weakness	109	90.8%
Jaundice	48	40%
Abdominal distension	106	88.3%
Blood in vomiting/black stool	44	37.5%
Altered sensorium/disorientation	30	25%
Fever	30	25%
Fatigue	63	52.5%
Easy bruising	97	80.8%
Loss of appetite	58	48.3%
Nausea	58	48.3%
Swelling of legs	12	10%
Weight loss	15	12.5%

**Figure 1:**
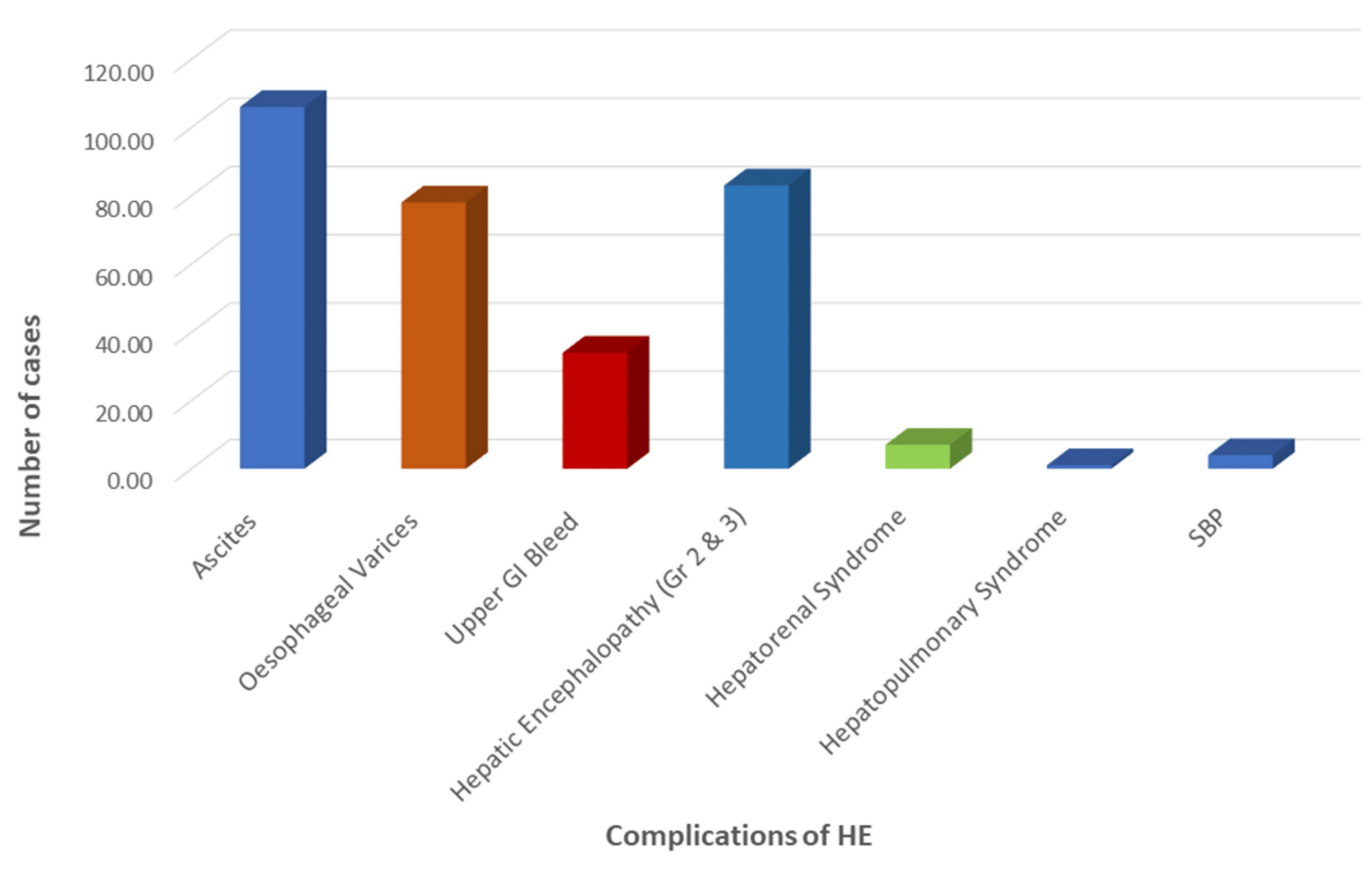
Distribution of complications in the study population (n=120) Abbreviations: SBP, spontaneous bacterial peritonitis; GI, gastrointestinal.

Ascites was the most frequently observed complication, affecting 106 patients (88.3%). Grade 3 ascites occurred in 70 cases (58.3%), while mild to moderate ascites was observed in 38 cases (31.8%; Figure 1). HE was the second most common complication, seen in 83 patients (69.2%), with grades 1, 2, and 3 encephalopathy occurring in 49 (40.8%), 17 (14.2%), and 17 (14.2%) cases, respectively. None of the patients experienced grade 4 encephalopathy. Oesophageal varices were detected in 78 cases (65%), with overt upper gastrointestinal bleeding occurring in 34 cases (23.3%). Infective complications, such as spontaneous bacterial peritonitis, were uncommon and observed in only four patients (3.3%). Hepatopulmonary syndrome was the least common complication, occurring in one patient (0.83%).

The mean CTP score for the study population was 9.68 ± 2.11, with values ranging from 5 to 15. Most patients (n=62, 51.7%) were classified as CTP Class B, followed by Class C (n=55, 45.8%) and Class A (n=3, 2.5%), as shown in Table 6.

**Table 6:** CTP Score level distribution in the study population (n=120) Abbreviations: CTP, Child-Turcotte-Pugh.

CTP Class	Number	Percentage
A	3	2.5%
B	62	51.7%
C	55	45.8%
Total	120	100%

**Figure 2:**
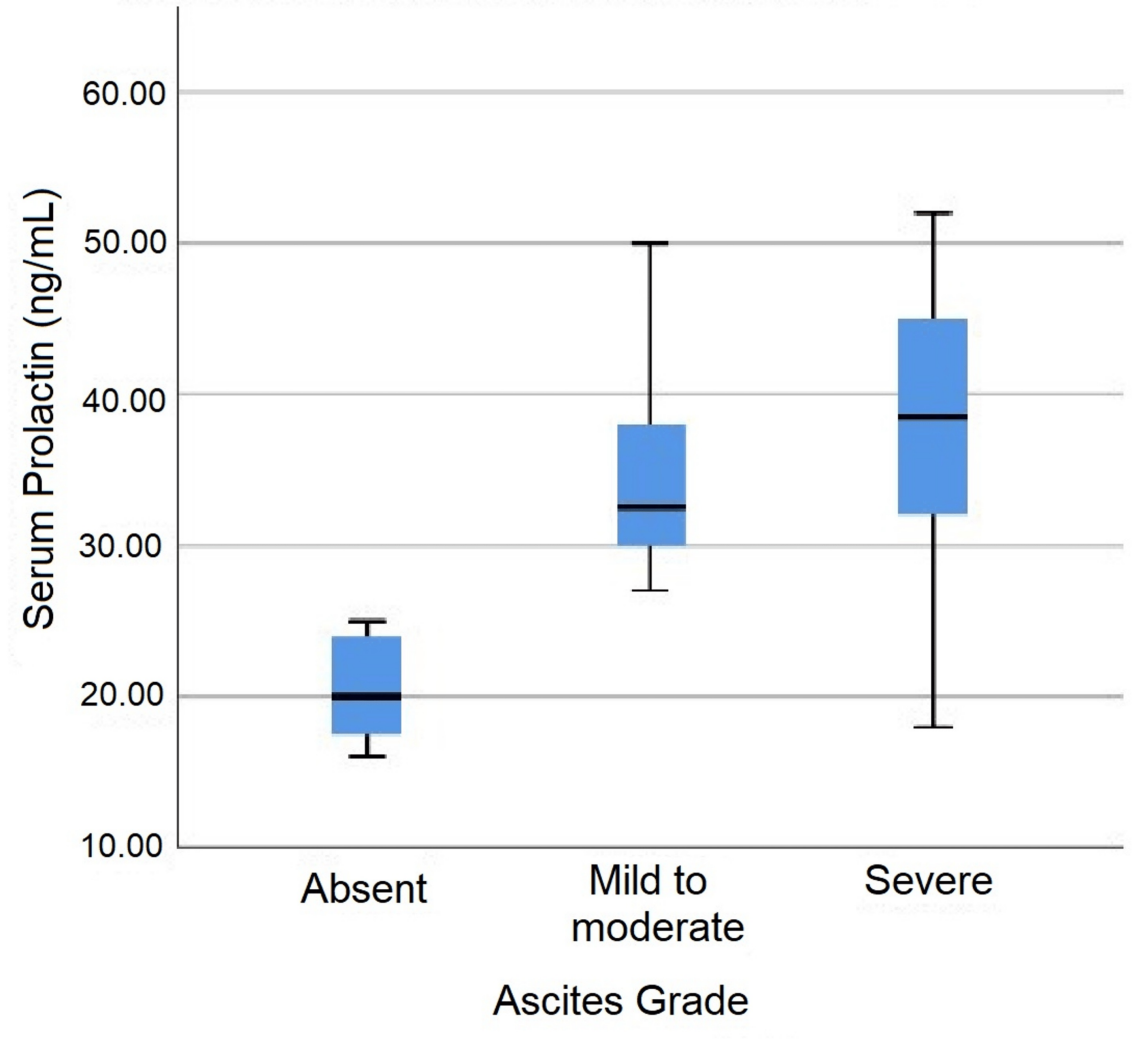
Independent-Samples Kruskal-Wallis Serum prolactin across grades of ascites

The mean serum prolactin level was 35.3 ± 9.25 ng/mL, with values ranging from 16 to 52 ng/mL. Most patients (n=76, 63.33%) had prolactin levels between 20 and 39 ng/mL, as summarized in Table 7. Serum prolactin levels demonstrated significant correlations with clinical components of the CTP score (Figure 2). Patients without ascites had a mean prolactin level of 20.58 ± 3.53 ng/mL, while those with mild to moderate ascites had 34.79 ± 7.13 ng/mL, and those with severe ascites had 38.10 ± 8.52 ng/mL. This difference was statistically significant (p < 0.001). Similarly, prolactin levels were significantly associated with HE severity. Patients without HE had a mean prolactin level of 28.8 ± 6.35 ng/mL, while levels increased progressively with grades 1, 2, and 3 encephalopathy (34.53 ± 9.04 ng/mL, 43.71 ± 5.61 ng/mL, and 43.2 ± 5.45 ng/mL, respectively, p < 0.001).

**Table 7:** Serum prolactin level in the study population (n=120)

Serum prolactin level (ng/mL)	Number	Percentage
<20	7	5.8%
20-39	76	63.3%
40-60	37	30.8%
Total	120	100%

**Figure 3:**
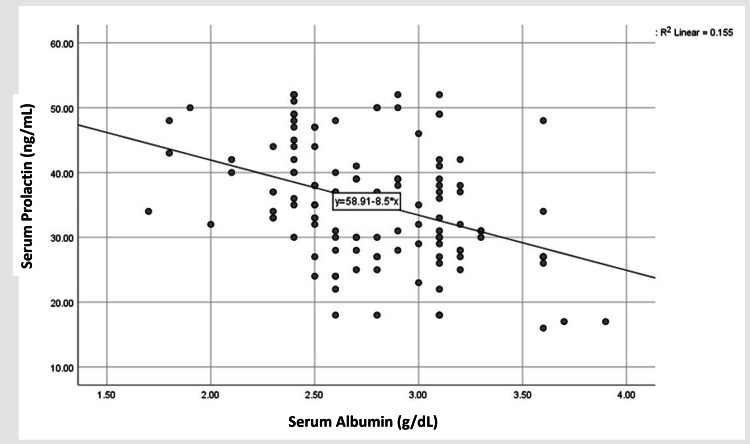
Correlation of serum prolactin level with serum albumin (n=120)

**Figure 4:**
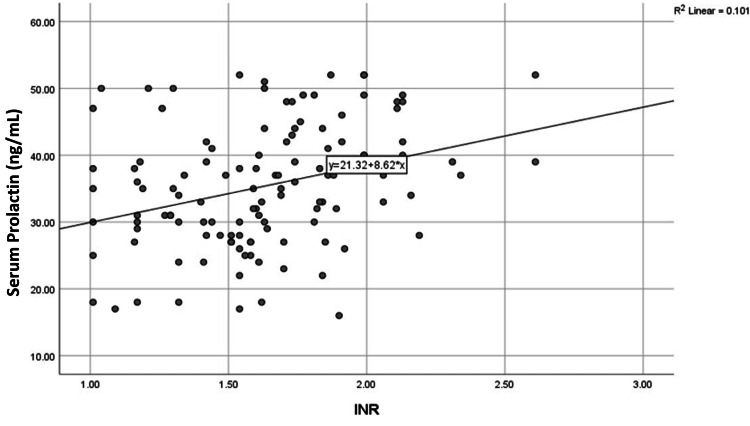
Correlation of serum prolactin level with INR (n=120) Abbreviation: INR, international normalized ratio

**Figure 5:**
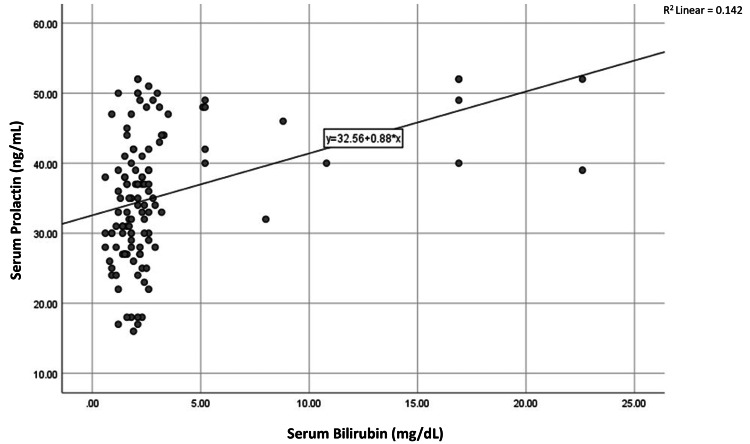
Correlation of serum prolactin level with serum bilirubin (n=120)

**Figure 6:**
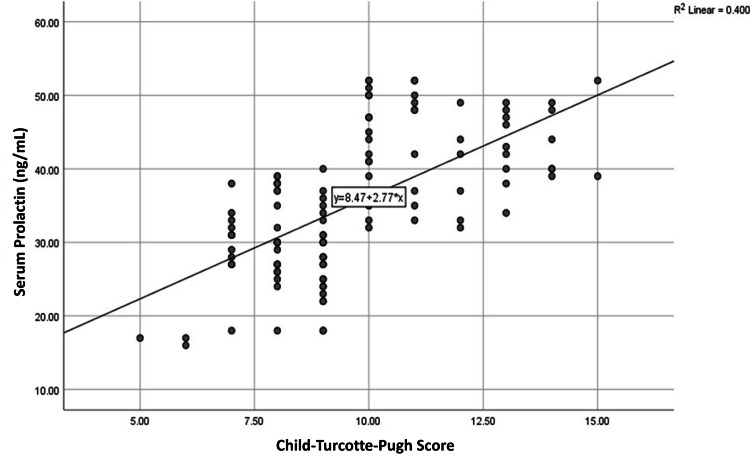
Correlation of serum prolactin with CTP score (n=120) Abbreviation: CTP, Child-Turcotte-Pugh

Parametric components of the CTP score also showed significant correlations with serum prolactin levels. We found a negative correlation with serum albumin (R = -0.393, p < 0.001) and positive correlations with INR (R = +0.317, p < 0.001) and serum bilirubin (R = +0.377, p < 0.001). Scatter plots illustrating these relationships are presented in Figures 3-6.

**Figure 7:**
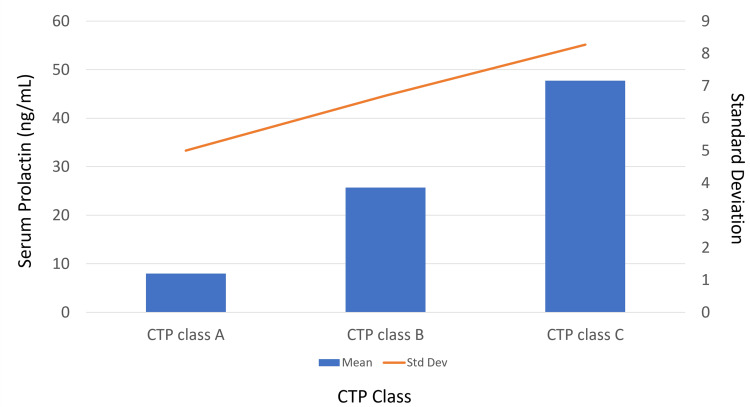
Comparison of serum prolactin level with CTP class in the study population (n=120) Abbreviations: CTP, Child-Turcotte-Pugh; Std Dev, standard deviation.

The mean serum prolactin level was significantly higher in patients classified as CTP Class C (47.91 ± 8.3 ng/mL) compared to Class B (25.8 ± 6.7 ng/mL) and Class A (8.0 ± 5.0 ng/mL; Figure 7). Differences between Class A and Class B were not statistically significant, but significant differences were observed between Class A and Class C and between Class B and Class C (Table 8; p < 0.001).

**Table 8:** Comparison of serum prolactin between CTP class in the study population (n=120) a- Independent sample Kruskal-Wallis test Abbreviations: CTP, Child-Turcotte-Pugh.

Sample 1-Sample 2	Test Statistic	Standard Error	Standard Test Statistic	P-Value^a^
A-B	-35.718	20.546	-1.738	0.082
A-C	-87.373	20.606	-4.240	0.000
B-C	-51.655	6.438	-8.024	0.000

**Figure 8:**
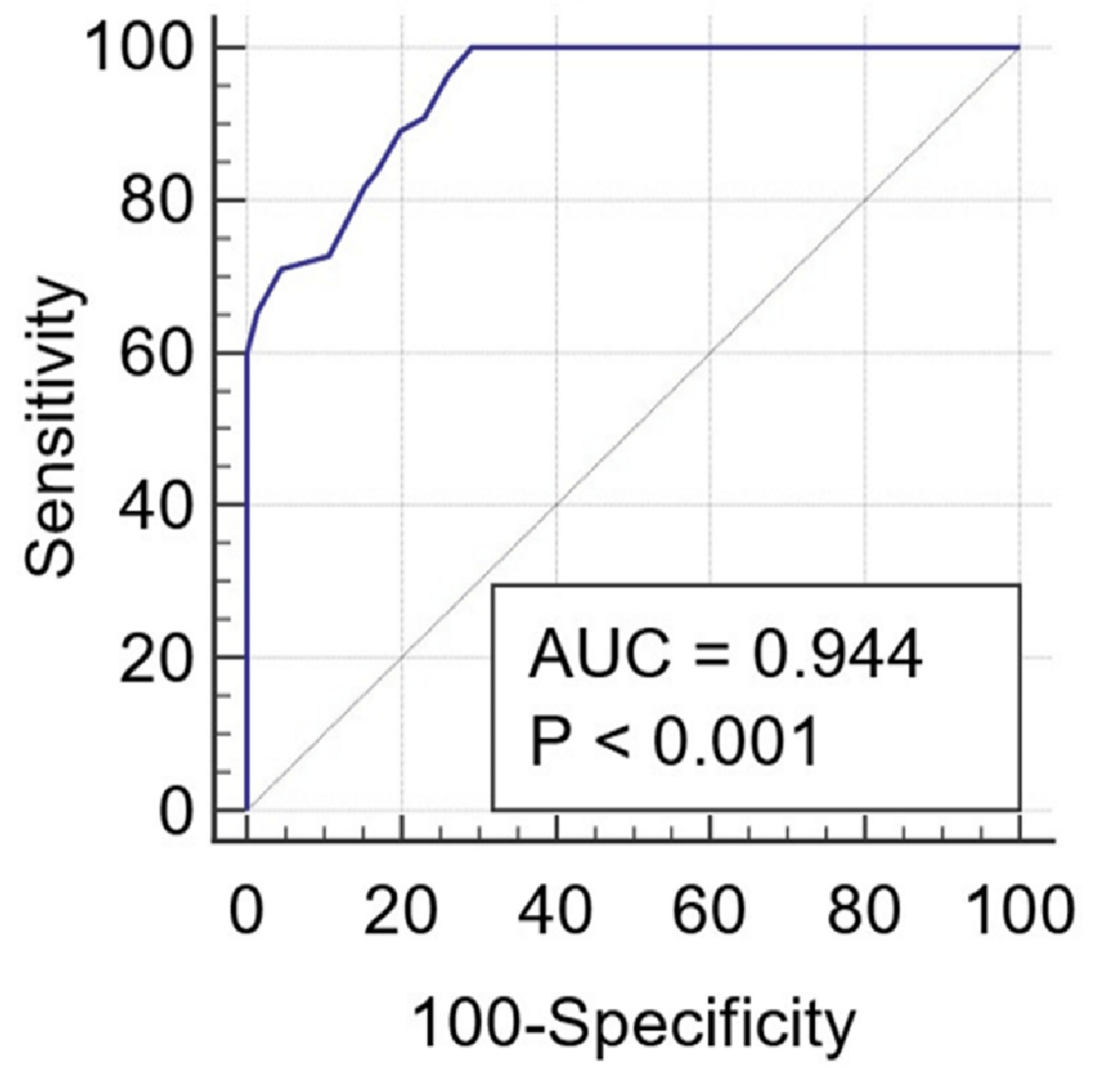
ROC graph of serum prolactin level with severity of CLD (CTP grade C – severe) Abbreviations: AUC, area under the curve; ROC, receiver operating characteristic; CLD, chronic liver disease; CTP, Child-Turcotte-Pugh.

ROC curve analysis revealed that serum prolactin is a strong predictor of severe CLD (CTP Class C), with an AUC of 0.944 (p < 0.001). A prolactin threshold of >35 ng/mL was associated with 83.64% sensitivity and 83.08% specificity for predicting severe CLD (Figure 8).

**Figure 9:**
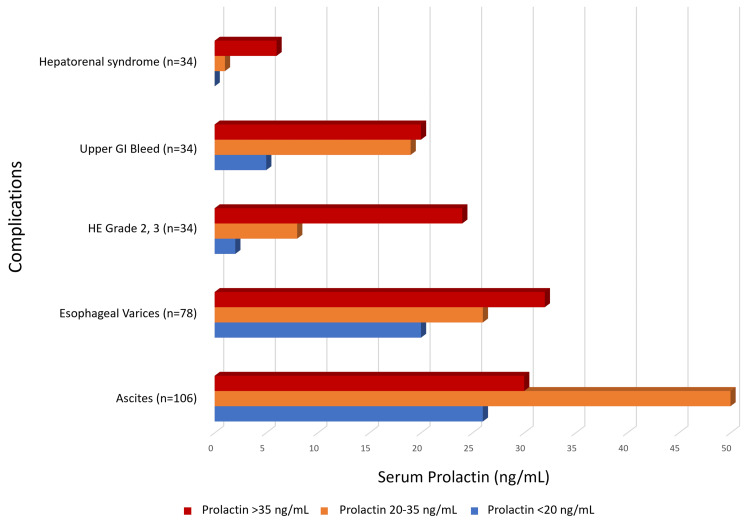
Correlation of serum prolactin level with various complications in the study population (n=120) Abbreviations: GI, gastrointestinal; HE, hepatic encephalopathy.

The distribution of complications across prolactin categories (<20 ng/mL, 20-35 ng/mL, and >35 ng/mL) is depicted in Figure 9, showing a higher incidence of complications with increasing prolactin levels.

Our study had a mortality rate of 3.33%, with four deaths occurring during the study period. Patients who died had significantly higher serum prolactin levels than survivors (p < 0.001). This relationship is detailed in Table 9. Also, haemoglobin, platelet count, serum proteins, and prothrombin time significantly correlated with serum prolactin levels, as shown in Table 10.

**Table 9:** Correlation of serum prolactin with mortality (n=120)

Group Statistics	Death	N	Mean	Standard Deviation	Standard Error Mean
Serum Prolactin	Yes	4	51.0000	1.15470	0.57735
	No	116	34.7586	8.92151	0.82834

**Table 10:** Serum prolactin correlation with all other cofactors in the study population (n=120) a-Pearson’s correlation coefficient Abbreviations: Hb, haemoglobin; WBC, white blood cell; BMI, body mass index.

Cofactors	Correlation R	P-Value^a^
Hb (g%)	-0.311	0.001
Platelet count (×1000)	-0.332	0.000
WBC count (/cu mm)	-0.075	0.416
Serum Protein (g/dl)	-0.196	0.032
Serum Globulin (g/dl)	0.097	0.294
Serum Creatinine (mg/dl)	-0.008	0.931
Prothrombin time (sec)	0.317	0.000
Weight (Kg)	0.160	0.080
Height (m)	0.060	0.518
BMI (kg/m^2^)	0.105	0.254
Waist circumference (cm)	0.034	0.714

## Discussion

CLD results from various aetiologies or long-term exposure to damaging factors, causing irreversible liver damage in the form of cirrhosis. The extent of morphological changes depends on the underlying cause and stage of the disease. This variability contributes to a variety of clinical presentations, including asymptomatic states and life-threatening complications. Hormonal dysregulation is a well-known feature of CLD, with defective secretion and abnormal feedback mechanisms contributing to the pathology [4]. Given that prolactin, a hormone implicated in various physiological processes, has potential as a marker for predicting complications in CLD, we conducted this study to evaluate the relationship between serum prolactin levels and the severity of CLD, via the CTP scoring system.

Age and sex

The mean age of the study population was 47.3 ± 10.4 years, with a male-to-female ratio of 6.5:1, indicating a clear male predominance. This observation aligns with findings from Waseem et al., who reported a mean age of 48.3 ± 12.08 years and a male predominance of 80.3% in their study [14]. Similarly, Punekar et al. reported a mean age of 44.9 ± 12.8 years and a male predominance of 86.7% in their population from central India [15]. Balakrishnan et al. observed that 75% of their participants were aged 40 to 50, with 83% male [16]. The higher prevalence of CLD in men is likely related to a higher incidence of alcohol consumption in men compared to women, contributing to the development of liver disease.

Aetiology of chronic liver disease

Alcohol consumption was the most common cause of cirrhosis in this study, accounting for 62.5% of cases, followed by MASH (9.2%) and hepatitis B virus infection (8.3%). Cryptogenic causes were observed in a small proportion of cases. Similar trends were noted by Punekar et al., who found alcohol to be the predominant aetiology in 55% of their study population, followed by hepatitis B in 18.3% [15]. Balakrishnan et al. reported alcohol-related cirrhosis in 73% of cases, hepatitis B in 9%, and hepatitis C in 5% [16]. However, Hong et al. reported hepatitis B virus as the most common cause of liver cirrhosis (56%), followed by alcohol use (21.7%) [17]. These findings highlight regional differences in the etiological spectrum of CLD.

Clinical features and complications of chronic liver disease

Generalized weakness and abdominal distension were our study’s most common presenting symptoms. Common complications included ascites, oesophagal varices, upper gastrointestinal bleeding, and HE grades 2 and 3. Most patients presented with advanced disease. Similar findings were reported by Punekar et al., where 90% of patients had ascites, 43.3% had esophageal varices, and 18.3% had grades 2 or 3 HE [15]. Notably, the current study did not include any cases of grade 4 HE, whereas Punekar et al. observed this in 11.7% of cases. Animesh et al. reported ascites in 98.5% of cases, oesophagal varices in 62.8%, upper gastrointestinal bleeding in 44.3%, and HE in 71.4% [18]. Balakrishnan et al. reported similar findings, with 88.3% of patients presenting with ascites and 65% with oesophageal varices, including portal gastropathy [16].

CTP score in the study population

The mean CTP score in this study was 9.7 ± 2.1. Most patients were classified as Class B, followed by Class C. Similar distributions were reported by Punekar et al., with 60% of their patients in Class B and 31.7% in Class C [15]. In contrast, Balakrishnan et al. observed a higher proportion of Class C cases (50%), with 40% in Class B and 10% in Class A [16]. Animesh et al. reported that 78.6% of their patients were in Class C, indicating significant disease severity in their cohort [18]. Velissaris et al. found that 42.9% of cases belonged to Class C, followed by 34.3% in Class A, showing a distinct distribution compared to the current study [19].

Serum prolactin levels

The mean serum prolactin level in the current study was 35.3 ± 9.3 ng/mL, with values ranging from 16 to 52 ng/mL. Animesh et al. reported a comparable mean prolactin level of 37.9 ± 6.6 ng/mL in their population [18]. However, lower mean prolactin levels were observed in studies by Punekar et al. (18.1 ± 11.3 ng/mL) and Khalil et al. (18.76 ± 9.14 ng/mL) [15,20]. Approximately 92% of patients in our study had elevated prolactin levels (using a cut-off of 19 ng/mL), compared to 73.3% in the study by Balakrishnan et al. [16]. These findings suggest that serum prolactin levels are commonly elevated in patients with CLD and may serve as a useful marker for disease severity.

Serum prolactin and CTP class

Serum prolactin levels showed a significant association with the CTP classification, as determined by the Kruskal-Wallis test (p < 0.001). Consistent with our findings, Balakrishnan et al. reported elevated serum prolactin levels with increasing CTP class (r = 0.787, p = 0.0095). They noted serum prolactin values >35 ng/mL in patients with CTP Class C, while nearly all Class A patients had normal levels, except one outlier [16]. Similarly, Patel et al. identified a strong positive correlation between serum prolactin levels and cirrhosis severity (r = 0.43, p = 0.004) [21]. Arafa et al. also observed significant increases in serum prolactin levels across CTP Classes A, B, and C (p = 0.023, p = 0.000, and p = 0.007, respectively) [22]. Animesh et al. further demonstrated that Class C patients (78.5%) had a mean prolactin level of 43.6 ng/mL, whereas all Class A patients exhibited normal prolactin levels (r = 0.83, p < 0.001) [18].

ROC analysis of serum prolactin and severe cirrhosis (CTP Class C)

ROC curve analysis in this study revealed an AUC of 0.944 (p < 0.001) for serum prolactin as a predictor of severe cirrhosis (CTP Class C). A serum prolactin cut-off >35 ng/mL demonstrated 83.64% sensitivity and 83.08% specificity. Similarly, Waseem et al. reported sensitivity and specificity values of 81.6% and 91.2%, respectively, for serum prolactin in predicting severe CTP scores [14]. Vemanamanda et al. observed an AUC of 0.845, with a prolactin cut-off >39.5 ng/mL yielding 82.61% sensitivity, 73.91% specificity, a negative predictive value of 89.47%, and an overall diagnostic accuracy of 76.81% (95% CI: 65.09%-86.13%) [23].

Serum prolactin levels with individual complications of CLD

This study demonstrated significantly higher serum prolactin levels in patients with more severe ascites (p < 0.001). Vemanamanda et al. also found significant differences in prolactin levels across ascites severity categories, with median levels of 58.00 ng/mL for severe ascites, 52.00 ng/mL for moderate ascites, 43.50 ng/mL for mild ascites, and 27.00 ng/mL for patients without ascites (p < 0.001) [23]. In contrast, Khalil et al. [20] and Punekar et al. [15] found no significant association between prolactin levels and ascites severity.

A significant correlation between serum prolactin levels and HE severity was observed in this study (p < 0.001). This aligns with findings from Arafa et al. [22] and Vemanamanda et al. [23], who reported higher serum prolactin levels across all HE grades than patients without HE. Khalil et al. also noted a strong positive correlation between serum prolactin and HE grades (r = 0.71, p < 0.001) [20]. However, Punekar et al. found no significant association between serum prolactin and HE [15].

Elevated serum prolactin levels were found in 76% of patients with oesophageal varices in this study. Similarly, Balakrishnan et al. reported elevated prolactin levels in 60.7% of patients with oesophageal varices [16]. Vemanamanda et al. observed higher median prolactin levels in patients with varices (46.00 ng/mL) compared to those without varices (35.00 ng/mL) [23]. Moreover, 90% of patients with upper gastrointestinal bleeding had markedly elevated serum prolactin levels in studies by Balakrishnan et al. [16] and Arafa et al. [22].

Serum prolactin and other lab parameters

We found significant correlations between serum prolactin levels and laboratory parameters, including haemoglobin, platelet count, serum bilirubin, prothrombin time, and serum albumin (all p < 0.001). Serum prolactin levels were negatively correlated with serum albumin and platelet count but positively correlated with serum bilirubin and prothrombin time. Khalil et al. reported similar findings, with mean albumin levels of 3.08 ± 0.85 g/dL, mean total bilirubin levels of 2.6 ± 1.3 mg/dL, and mean prothrombin time of 8.9 ± 5.54 seconds [20]. Sakhnani also found a significant positive correlation between serum prolactin and serum bilirubin (r = +0.4041, p < 0.003) and INR (r = +0.7637, p < 0.0001). Negative correlations were noted with serum albumin (r = -0.7412, p < 0.0001) and platelet count (r = -0.8379, p < 0.0001) [24]. Punekar et al. similarly reported significant positive correlations between serum prolactin levels, bilirubin levels (rs = 0.372, p = 0.003), and prothrombin time (rs = 0.490, p < 0.0001). However, they found no association between serum prolactin and albumin or creatinine [15].

Mortality in the study population

This study identified significantly higher serum prolactin levels in patients who died compared to survivors (p < 0.001). Jha et al. observed that mortality was higher in patients with serum prolactin levels >50 ng/mL, with 47.8% of these patients dying compared to 33.3% of those with levels <50 ng/mL (p < 0.05) [25]. Giri et al. also reported increased mortality among patients with serum prolactin levels >50 ng/mL [26]. Punekar et al. highlighted the utility of serum prolactin as a prognostic indicator for predicting complications and mortality in liver cirrhosis [15]. These findings reinforce the importance of serum prolactin as a marker of disease severity and prognosis in CLD.

Limitations of the study

Our study had several limitations. Our sample size was relatively small, which may limit the generalizability of the findings. Second, the relationship between serum prolactin levels and complications such as spontaneous bacterial peritonitis and hepatopulmonary syndrome could not be evaluated due to the small number of cases with these conditions. Lastly, the study did not assess the correlation between the degree of serum prolactin elevation and the specific aetiologies of CLD.

## Conclusions

This study demonstrated that serum prolactin levels in patients with CLD serve as a reliable marker of disease severity, as they strongly correlate with the CTP grading system. Furthermore, serum prolactin levels were significantly elevated in patients with complications such as HE, ascites, and oesophageal varices. Higher prolactin levels were associated with more severe grades of encephalopathy and increased mortality. Based on these findings, serum prolactin can be considered a valuable prognostic marker in patients with CLD. It offers a non-invasive approach to identifying patients at high risk of complications and mortality, thus aiding in early intervention and management strategies.
